# IgG4-related sclerosing cholangitis: navigating diagnostic dilemmas and the challenge of relapse

**DOI:** 10.3389/fmed.2026.1732637

**Published:** 2026-02-05

**Authors:** Xiangxiang Ren, Xiaoshi Jin, Litao Liu, Meng Zhang

**Affiliations:** 1 Department of General Surgery, Affiliated Hospital of Hebei University, Baoding, Hebei, China; 2 Department of Dermatology, Affiliated Hospital of Hebei University, Baoding, Hebei, China

**Keywords:** autoimmune pancreatitis, corticosteroid therapy, diagnosis, differential diagnosis, IgG4-related cholangitis, IgG4-related sclerosing cholangitis, inebilizumab, relapse

## Abstract

**Background:**

Immunoglobulin G4-related sclerosing cholangitis (IgG4-SC), also termed IgG4-related cholangitis (IRC), is a challenging immune-mediated biliary disease, frequently mimicking malignancies such as cholangiocarcinoma (CCA) or other sclerosing cholangitides like primary sclerosing cholangitis (PSC). Accurate diagnosis is critical to avoid unnecessary surgical interventions.

**Objective:**

This mini-review aims to synthesize the most current evidence on the pathogenesis, diagnostic pitfalls, and management strategies for IgG4-SC, with a focused discussion on overcoming diagnostic dilemmas and addressing the significant challenge of disease relapse.

**Key findings:**

The pathogenesis of IgG4-SC involves a complex interplay of genetic predisposition, environmental triggers (e.g., industrial vapors, dust, gases, fumes, and asbestos), and dysregulated adaptive immunity. A distinctive CD4+ T-cell response, dominated by T-helper 2 (Th2), follicular helper T (Tfh) cells, and regulatory T cells (Tregs), drives B-cell activation, oligoclonal expansion of IgG4+ plasmablasts, and progressive fibrosis. Notably, the discovery of IgG4/IgG1 autoantibodies against annexin A11 and laminin 511-E8 has provided insight into potential direct pathogenic mechanisms. Diagnosis relies on a multimodal approach integrating clinical presentation, characteristic imaging findings, elevated serum IgG4 levels (with levels >2× ULN being suggestive, and >4× ULN being highly specific), the IgG4/IgG1 ratio (>0.24), other organ involvement (notably type 1 autoimmune pancreatitis, AIP), supportive histopathology, and a rapid response to corticosteroid therapy. Despite high initial response rates to steroids, relapse occurs in 30%–50% of patients. Maintenance therapy with steroid-sparing immunomodulators (e.g., azathioprine, mycophenolate mofetil) or B-cell depleting agents such as rituximab is often required. The anti-CD19 monoclonal antibody inebilizumab has emerged as a potent new option for maintaining remission.

**Conclusion:**

Maintaining a high index of clinical suspicion for IgG4-SC is essential in patients with obstructive jaundice and biliary strictures. Future efforts should focus on validating specific biomarkers (e.g., circulating plasmablasts, autoantibody profiles) and developing evidence-based protocols for long-term management to prevent fibrotic complications and reduce the relapse rate.

## 1 Introduction

Immunoglobulin G4-related sclerosing cholangitis (IgG4-SC), or IgG4-related cholangitis (IRC), is the biliary manifestation of a multi-system fibroinflammatory condition termed IgG4-related disease (IgG4-RD). Initially described in the context of type 1 autoimmune pancreatitis (AIP), it is now recognized as a principal cause of benign biliary strictures that can be radiologically indistinguishable from cholangiocarcinoma (CCA) (1, 2). Historically, misdiagnosis has led to unnecessary pancreaticoduodenectomy or liver resection (3), underscoring the critical importance of accurate diagnosis. The disease is pathologically characterized by lymphoplasmacytic infiltration, storiform fibrosis, obliterative phlebitis, and a significant increase in IgG4-positive plasma cells within the bile duct wall (4).

Although corticosteroid therapy is highly effective in the inflammatory phase, disease relapse is common, and long-term sequelae from progressive fibrosis pose a significant management challenge (5). This review provides a contemporary overview of the immunopathogenesis, clinical and diagnostic features, differential diagnosis, and treatment paradigms of IgG4-SC, with particular emphasis on navigating diagnostic dilemmas and strategies to mitigate relapse. It highlights ongoing controversies and future directions, incorporating insights from the latest international guidelines and clinical trials (6, 7).

## 2 Unraveling the pathogenesis: a complex immune dysregulation

The precise etiology of IgG4-SC remains elusive; however, current evidence suggests an antigen-driven process in genetically susceptible individuals, often triggered by environmental exposures, leading to a distinctive CD4+ T-cell and B-cell response (8, 9).

### 2.1 Genetic predisposition and environmental triggers

Genome-wide association studies have identified several risk loci for IgG4-RD, including HLA-DRB1 and non-HLA genes such as FCCR2B and CTL44, indicating roles in antigen presentation and immune regulation (10, 11). Notably, long-term exposure to occupational toxins–specifically industrial vapors, dust, gases, fumes (VDGF), and asbestos–has been established as a significant environmental risk factor, which may contribute to the strong male predominance in IgG4-SC (9, 12). Cigarette smoking has also been associated with an increased risk of IgG4-RD, particularly retroperitoneal fibrosis (13).

### 2.2 The central and diversified role of T lymphocytes

A dominant T-helper 2 (Th2) and regulatory T cell (Treg) response is a hallmark of IgG4-SC (14). Th2 cytokines (IL-4, IL-5, IL-13) promote B-cell activation, isotype switching, and are implicated in associated eosinophilia. T follicular helper (Tfh) cells, particularly the Th2 subset, facilitate germinal center formation, B-cell differentiation, and IgG4 production (15). T peripheral helper (Tph) cells are also increased and may promote ectopic lymphoid structure formation, specifically tertiary lymphoid structures (TLS), within lesions. Notably, a significant proportion of these Th2-polarized cells express the transcription factor GATA3 and also exhibit cytotoxic T lymphocyte (CTL) markers, blurring the traditional Th2/CTL dichotomy. These GATA3 + CTLs are clonally expanded within lesions and contribute directly to tissue damage via perforin and granzyme secretion, highlighting a unique pathogenic T-cell subset that combines helper and cytotoxic functions (16, 17). Concurrently, an abundance of Tregs (FOXP3+, CD4+, CD25+) within lesions overexpresses IL-10 and TGF-β. While IL-10 contributes to the IgG4 class switch, TGF-β is a potent pro-fibrotic cytokine, directly fueling the tissue fibrosis characteristic of advanced disease (14, 18).

### 2.3 B cells, plasmablasts and the dual nature of IgG4 antibodies

A robust, oligoclonal B-cell and plasmablast response is evident in active disease, serving as a sensitive biomarker (19, 20). Although tissue infiltration by IgG4-positive plasma cells is a pathological cornerstone, the role of the IgG4 antibody itself is complex. Its unique structural characteristic of “Fab-arm exchange” results in bispecific antibodies with poor immune complex-forming capacity, suggesting it may function as an “anti-inflammatory” antibody (21). However, a paradigm shift has occurred with the identification of specific autoantigens. IgG4 (and IgG1) autoantibodies against annexin A11 and laminin 511-E8 have been demonstrated to be pathogenic *in vitro* and in animal models, potentially by directly blocking the function of these critical proteins, thereby impairing cholangiocyte protective mechanisms (e.g., the “biliary bicarbonate umbrella”) and contributing to bile duct injury (22, 23). Thus, IgG4 may serve as both a marker of disease and, in specific contexts, a direct mediator of pathology.

### 2.4 Translational implications for diagnosis and therapy

Understanding this intricate immunopathology directly informs current challenges. The oligoclonal expansion of B-cells and plasmablasts underpins the use of B-cell depletion therapy (e.g., rituximab, inebilizumab) and supports the investigation of circulating plasmablasts as a biomarker for disease activity and relapse risk (24, 25). The identification of pathogenic autoantibodies opens avenues for developing antigen-specific diagnostic assays, though their clinical utility requires further validation (22, 23). Similarly, characterizing dominant T-cell subsets (Th2, Tfh, Tph) offers potential future targets for more precise immunomodulation.

## 3 Clinical presentation and diagnostic approach

### 3.1 Clinical, laboratory, and imaging features

The disease predominantly affects middle-aged to elderly males, who frequently present with painless obstructive jaundice, mimicking pancreatic or biliary malignancy (3, 24–26). Many patients have or will develop other organ involvement, most commonly type 1 AIP, but also retroperitoneal fibrosis, sclerosing sialadenitis, and dacryoadenitis (27, 28). Characteristic laboratory findings include elevated serum IgG4 levels (observed in approximately 80% of patients). The IgG4/IgG1 ratio (cut-off > 0.24) can significantly improve diagnostic specificity in cases with moderately elevated IgG4 (1–2× ULN) (29).

Imaging modalities, including CT, MRI/MRCP, and EUS, are crucial. Key features favoring IgG4-SC include long-segment (>3 cm), band-like strictures with concentric, symmetric wall thickening (>2.5 mm) and a smooth inner margin. In contrast, CCA often presents with shorter, asymmetric, mass-like thickening. The absence of significant upstream ductal dilation relative to the degree of stenosis can also be a clue. Magnetic resonance cholangiopancreatography (MRCP) is essential for mapping stricture morphology and distribution. Intraductal ultrasound (IDUS) features, such as circular symmetric wall thickness and a layered “sandwich” pattern, are highly suggestive of IgG4-SC (30, 31). Positron Emission Tomography (PET) with 18F-fluorodeoxyglucose (FDG-PET) can provide valuable functional information in IgG4-SC. PET often demonstrates diffusely increased FDG uptake along the involved bile ducts and in other affected organs (e.g., pancreas, salivary glands), which may help in assessing disease activity, identifying subclinical multi-organ involvement, and monitoring treatment response. However, it is not routinely required for diagnosis and should be interpreted alongside morphological imaging findings (32).

Immunoglobulin G4-related sclerosing cholangitis is classically classified into four types based on stricture location (Table 1) (33). This classification has practical utility, as different types mimic different diseases (e.g., Type 1 vs. pancreatic cancer, Type 4 vs. PSC or hilar CCA).

**TABLE 1:** Cholangiographic classification of IgG4-SC [Nishino T et al. (33)].

Type	Description of stricture location	Main differential diagnosis
1	Distal third of the common bile duct	Pancreatic cancer, chronic pancreatitis
2	Intrahepatic ducts, without hilar involvement	Not specified
3	Hilar and distal common bile duct	Hilar cholangiocarcinoma
4	Strictures involving both hilar and intrahepatic ducts	Primary sclerosing cholangitis, hilar cholangiocarcinoma

### 3.2 Diagnostic criteria and the role of histology

The HISORt criteria remain a comprehensive diagnostic system, integrating Histology, Imaging, Serology, Other organ involvement, and Response to corticosteroid therapy (34). A definitive diagnosis can be established with characteristic histology [requiring at least two of the following three features: dense lymphoplasmacytic infiltrate, storiform fibrosis, and obliterative phlebitis, supported by >10 IgG4+ plasma cells/HPF in biopsy or >50/HPF in resection specimens (4)] or a combination of typical imaging, elevated serum IgG4 (>2× ULN), and evidence of other organ involvement.

It is critically important to note that an elevated serum IgG4 level alone is not diagnostic. Levels can be modestly elevated in 10%–15% of patients with PSC or CCA (31–36). While a level exceeding four times the upper limit of normal (ULN) is highly specific for IgG4-SC, its sensitivity is low. Therefore, serum IgG4 must always be interpreted within the entire clinical context. Updated international guidelines emphasize a structured diagnostic workflow to categorize patients into “definitive” or “probable” IgG4-SC, with the latter group warranting a diagnostic steroid trial only after malignancy has been rigorously excluded (6, 7).

**FIGURE 1:**
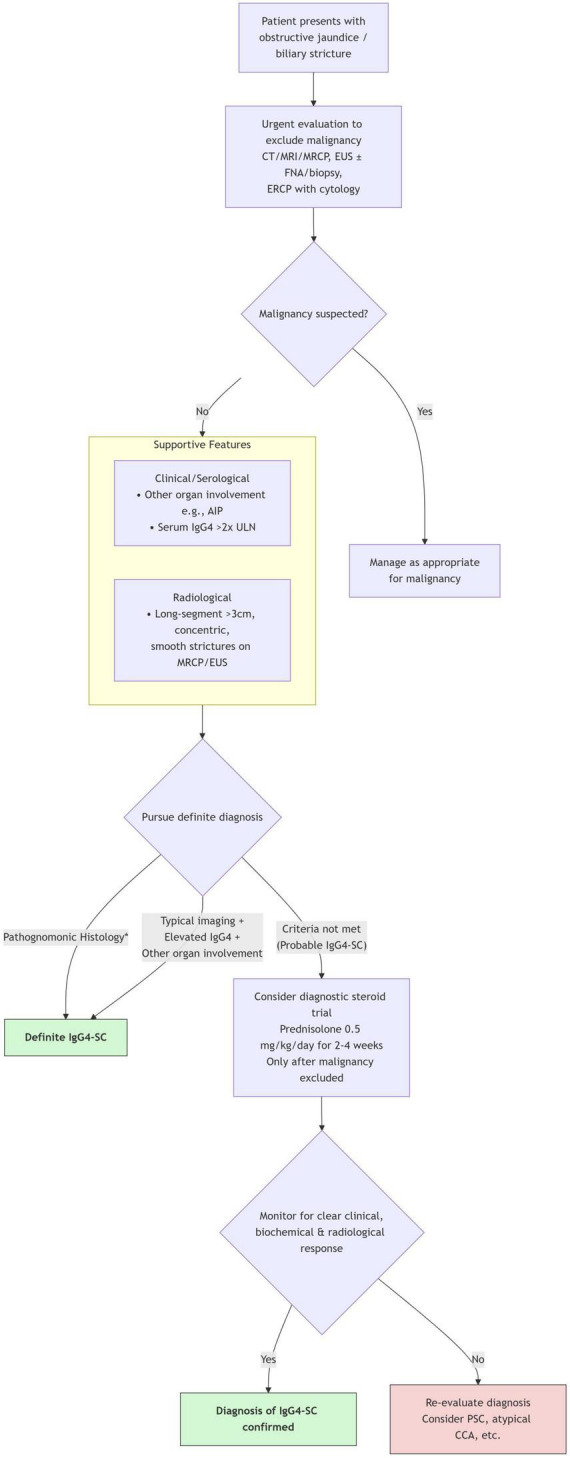
Diagnostic algorithm for suspected IgG4-sclerosing cholangitis. *Pathognomonic histology from bile duct/liver biopsy requires ≥2 of: dense lymphoplasmacytic infiltrate, storiform fibrosis, obliterative phlebitis, and >10 IgG4+ plasma cells per high-power field. AIP, autoimmune pancreatitis; CCA, cholangiocarcinoma; ERCP, endoscopic retrograde cholangiopancreatography; EUS, endoscopic ultrasound; FNA, fine-needle aspiration; HPF, high-power field; MRCP, magnetic resonance cholangiopancreatography; PSC, primary sclerosing cholangitis; ULN, upper limit of normal.

Given the multifaceted nature of IgG4-SC, no single test is pathognomonic. Therefore, a structured, multimodal diagnostic algorithm is essential to integrate the clinical, serological, radiological, and histological features discussed above. Figure 1 provides a practical diagnostic workflow that synthesizes these components, guiding clinicians from the initial presentation of a biliary stricture toward a definitive or probable diagnosis of IgG4-SC, while emphasizing the critical first step of ruling out malignancy. The subsequent section delves into the practical application of this workflow in differentiating IgG4-SC from its key mimics.

## 4 Navigating the differential diagnosis

The differentiation of IgG4-SC from its mimics is a common clinical dilemma, necessitating a multimodal approach.

### 4.1 IgG4-SC vs. cholangiocarcinoma (CCA)

This distinction is paramount. Clinically, the presence of other IgG4-RD manifestations strongly favors IgG4-SC. Serologically, a serum IgG4 level >2× ULN supports IgG4-SC, and specificity increases significantly at levels >4× ULN. The IgG4/IgG1 ratio is also useful (27–29). On imaging, IgG4-SC often presents with longer, smoother strictures and more diffuse, symmetric bile duct wall thickening compared to the asymmetric, mass-like thickening often seen in CCA. Histologically, bile duct biopsies showing a characteristic lymphoplasmacytic infiltrate with abundant IgG4-positive plasma cells and absence of atypia support IgG4-SC. Finally, a carefully monitored 2- to 4-weeks diagnostic corticosteroid trial can be employed, which typically leads to symptomatic, biochemical, and radiological improvement in IgG4-SC but not in CCA; this approach, however, should only be considered after malignancy has been rigorously investigated and excluded (6).

### 4.2 IgG4-SC vs. primary sclerosing cholangitis (PSC)

The distinction between these two sclerosing cholangitides is critical. Key differences include the older age of onset and male predominance in IgG4-SC, the strong association of PSC with inflammatory bowel disease (IBD), the presence of other organ involvement in IgG4-SC, and the dramatic response of IgG4-SC to corticosteroid therapy, which is ineffective in PSC. Imaging features such as the absence of “pruning” and the presence of long, band-like strictures also favor IgG4-SC (37). Novel serum metabolomic panels, which profile small-molecule metabolites to distinguish disease states, show promise in improving this differentiation (38).

## 5 Treatment strategies and the challenge of relapse

### 5.1 Induction of remission

Corticosteroids constitute the first-line therapy for active IgG4-SC. A standard induction regimen is oral prednisolone (0.5–0.6 mg/kg/day) for 2–4 weeks, followed by a gradual taper over 2–3 months (5, 6). Rapid improvement in symptoms, biochemistry, and imaging is typically observed and serves as a diagnostic confirmation. Medium-dose prednisone has been shown to be as effective as high dose for remission induction (39). For patients with severe obstructive jaundice, biliary stenting via ERCP may be necessary prior to initiating steroid therapy.

### 5.2 Managing relapse and maintenance therapy

Disease relapse occurs in 30%–50% of patients, often during steroid taper or after discontinuation (5). Risk factors include proximal (hilar/intrahepatic) strictures, persistent bile duct wall thickening on imaging, and persistently elevated serum IgG4 (5, 40).

#### 5.2.1 Steroid-sparing immunomodulators

For relapsing disease, steroid dependence, or as part of an initial maintenance strategy, agents such as azathioprine (1.5–2.5 mg/kg/day) or mycophenolate mofetil are commonly employed. Combination therapy with glucocorticoids is associated with higher remission rates than steroids alone (40, 41).

#### 5.2.2 B-cell depleting therapy

The anti-CD20 monoclonal antibody rituximab is highly effective for inducing and maintaining remission, both in relapsing/refractory disease and as a first-line steroid-sparing agent (42, 43). More recently, the anti-CD19 monoclonal antibody inebilizumab demonstrated significant efficacy in reducing relapse risk in a phase 3 randomized controlled trial, confirming the central role of B-cell depletion (44). Beyond rituximab and inebilizumab, several novel agents are under investigation in ongoing clinical trials. These include other B-cell-targeting therapies, agents targeting specific cytokines or T-cell pathways, and antifibrotic compounds. The results of these trials are awaited to further expand the therapeutic arsenal for IgG4-SC (44, 45).

**FIGURE 2:**
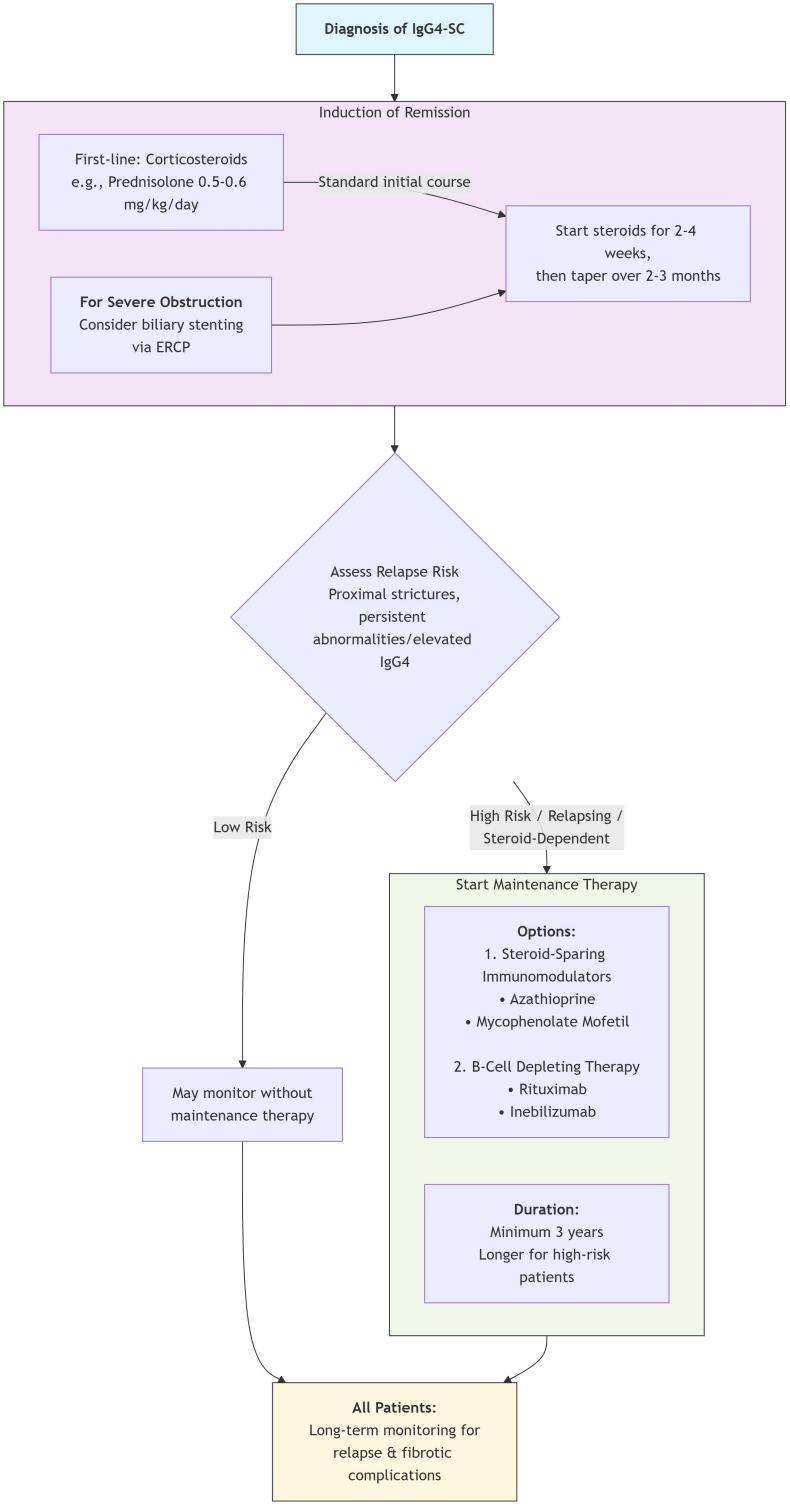
Treatment pathway for IgG4-sclerosing cholangitis. ERCP, endoscopic retrograde cholangiopancreatography; IgG4-SC, Immunoglobulin G4-related sclerosing cholangitis.

The decision regarding the duration of maintenance therapy is individualized, but current evidence supports a minimum of 3 years, with longer-term treatment considered for high-risk patients (6, 43). The overall strategy for induction and maintenance therapy, incorporating the options for steroid-sparing agents and B-cell depletion, is summarized in Figure 2.

## 6 Conclusion and future perspectives

Immunoglobulin G4-related sclerosing cholangitis is a unique biliary disease that sits at the intersection of hepatology, gastroenterology, rheumatology, and immunology. This review has emphasized the critical importance of recognizing its mimicker status to prevent misdiagnosis and unnecessary procedures. While significant progress has been made–particularly in elucidating its complex immune pathogenesis, identifying environmental triggers and specific autoantigens, and establishing multimodal diagnostic criteria–several challenges remain. The high relapse rate underscores the need for superior biomarkers (e.g., circulating plasmablasts, memory B cells) to predict disease course and guide maintenance therapy. Future research should be directed toward: (1) Identifying antigens: unraveling the initial disease-triggering antigens and their role in breaking immune tolerance. (2) Validating biomarkers: developing and validating non-invasive biomarkers for diagnosis, monitoring disease activity, and predicting relapse. (3) Optimizing treatment: conducting large, randomized controlled trials to establish optimal steroid-sparing regimens, define the long-term role of B-cell-targeted therapies, and explore novel strategies to reverse or halt established fibrosis.

## References

[1] Kamisawa T Nakazawa T Tazuma S Zen Y Tanaka A Ohara H Clinical practice guidelines for IgG4-related sclerosing cholangitis. *J Hepatobiliary Pancreat Sci*. (2019) 26:9–42. 10.1002/jhbp.596 30575336 PMC6590186

[2] Kersten R Trampert DC Herta T Hubers LM Maillette de Buy Wenniger LJ Verheij J IgG4-related cholangitis - a mimicker of fibrosing and malignant cholangiopathies. *J Hepatol*. (2023) 79:1502–23. 10.1016/j.jhep.2023.08.005 37598939

[3] Ghazale A Chari ST Zhang L Smyrk TC Takahashi N Levy MJ Immunoglobulin G4-associated cholangitis: clinical profile and response to therapy. *Gastroenterology*. (2008) 134:706–15. 10.1053/j.gastro.2007.12.009 18222442

[4] Deshpande V Zen Y Chan JK Yi EE Sato Y Yoshino T Consensus statement on the pathology of IgG4-related disease. *Mod Pathol*. (2012) 25:1181–92. 10.1038/modpathol.2012.72 22596100

[5] Hart PA Topazian MD Witzig TE Clain JE Gleeson FC Klebig RR Treatment of relapsing autoimmune pancreatitis with immunomodulators and rituximab: the Mayo Clinic experience. *Gut*. (2013) 62:1607–15. 10.1136/gutjnl-2012-302886 22936672

[6] European Association for the Study of the Liver. EASL Clinical Practice Guidelines on sclerosing cholangitis. *J Hepatol.* (2022) 77:761–806. 10.1016/j.jhep.2022.05.011 35738507

[7] Löhr JM Beuers U Vujasinovic M Alvaro D Frøkjær JB Buttgereit F European Guideline on IgG4-related digestive disease - UEG and SGF evidence-based recommendations. *United European Gastroenterol J*. (2020) 8:637–66. 10.1177/2050640620934911 32552502 PMC7437085

[8] Pillai S Perugino C Kaneko N. Immune mechanisms of fibrosis and inflammation in IgG4-related disease. *Curr Opin Rheumatol*. (2020) 32:146–51. 10.1097/BOR.0000000000000686 31842033

[9] Hubers LM Schuurman AR Buijs J Mostafavi N Bruno MJ Vermeulen RCH Blue-collar work is a risk factor for developing IgG4-related disease of the biliary tract and pancreas. *JHEP Rep*. (2021) 3:100385. 10.1016/j.jhepr.2021.100385 34816110 PMC8593662

[10] Terao C Ota M Iwasaki T Shiokawa M Kawaguchi S Kuriyama K IgG4-related disease in the Japanese population: a genome-wide association study. *Lancet Rheumatol*. (2019) 1:e14–22. 10.1016/S2665-9913(19)30006-2 38229354

[11] Ishikawa Y Terao C. Genetic analysis of IgG4-related disease. *Mod Rheumatol*. (2020) 30:17–23. 10.1080/14397595.2019.1621000 31104539

[12] de Buy Wenniger LJ Culver EL Beuers U. Exposure to occupational antigens might predispose to IgG4-related disease. *Hepatology*. (2014) 60:1453–4. 10.1002/hep.26999 24407836 PMC4258085

[13] Wallwork R Perugino CA Fu X Harkness T Zhang Y Choi HK The association of smoking with immunoglobulin G4-related disease: a case-control study. *Rheumatology*. (2021) 60:5310–7. 10.1093/rheumatology/keab172 33751033 PMC8783539

[14] Zen Y Fujii T Harada K Kawano M Yamada K Takahira M Th2 and regulatory immune reactions are increased in immunoglobin G4-related sclerosing pancreatitis and cholangitis. *Hepatology*. (2007) 45:1538–46. 10.1002/hep.21697 17518371

[15] Akiyama M Suzuki K Yasuoka H Kaneko Y Yamaoka K Takeuchi T. Follicular helper T cells in the pathogenesis of IgG4-related disease. *Rheumatology*. (2018) 57:236–45. 10.1093/rheumatology/kex171 28460058

[16] Mattoo H Mahajan VS Maehara T Deshpande V Della-Torre E Wallace ZS Clonal expansion of CD4(+) cytotoxic T lymphocytes in patients with IgG4-related disease. *J Allergy Clin Immunol*. (2016) 138:825–38. 10.1016/j.jaci.2015.12.1330 26971690 PMC5014627

[17] Perugino CA Kaneko N Maehara T Mattoo H Kers J Allard-Chamard H CD4+ and CD8+ cytotoxic T lymphocytes may induce mesenchymal cell apoptosis in IgG4-related disease. *J Allergy Clin Immunol*. (2021) 147:368–82. 10.1016/j.jaci.2020.05.022 32485263 PMC7704943

[18] Della-Torre E Lanzillotta M Doglioni C. Immunology of IgG4-related disease. *Clin Exp Immunol*. (2015) 181:191–206. 10.1111/cei.12641 25865251 PMC4516435

[19] Maillette de Buy Wenniger LJ Doorenspleet ME Klarenbeek PL Verheij J Baas F Elferink RP Immunoglobulin G4+ clones identified by next-generation sequencing dominate the B cell receptor repertoire in immunoglobulin G4 associated cholangitis. *Hepatology.* (2013) 57:2390–8. 10.1002/hep.26232 23300096

[20] Wallace ZS Mattoo H Carruthers M Mahajan VS Della Torre E Lee H Plasmablasts as a biomarker for IgG4-related disease, independent of serum IgG4 concentrations. *Ann Rheum Dis*. (2015) 74:190–5. 10.1136/annrheumdis-2014-205233 24817416 PMC4656194

[21] Rispens T Huijbers MG. The unique properties of IgG4 and its roles in health and disease. *Nat Rev Immunol*. (2023) 23:763–78. 10.1038/s41577-023-00871-z 37095254 PMC10123589

[22] Hubers LM Vos H Schuurman AR Erken R Oude Elferink RP Burgering B Annexin A11 is targeted by IgG4 and IgG1 autoantibodies in IgG4-related disease. *Gut*. (2018) 67:728–35. 10.1136/gutjnl-2017-314548 28765476

[23] Herta T Kersten R Chang JC Hubers L Go S Tolenaars D Role of the IgG4-related cholangitis autoantigen annexin A11 in cholangiocyte protection. *J Hepatol*. (2022) 76:319–31. 10.1016/j.jhep.2021.10.009 34718050 PMC10804347

[24] Lanzillotta M Ramirez GA Milani R Dagna L Della-Torre EB. cell depletion after treatment with rituximab predicts relapse of IgG4-related disease. *Rheumatology*. (2025) 64:2290–4. 10.1093/rheumatology/keae248 38781535 PMC11962880

[25] Khosroshahi A Wallace ZS Crowe JL Akamizu T Azumi A Carruthers MN International consensus guidance statement on the management and treatment of IgG4-related disease. *Arthritis Rheumatol*. (2015) 67:1688–99. 10.1002/art.39132 25809420

[26] Löhr JM Vujasinovic M Rosendahl J Stone JH Beuers U. IgG4-related diseases of the digestive tract. *Nat Rev Gastroenterol Hepatol*. (2022) 19:185–97. 10.1038/s41575-021-00529-y 34750548

[27] Hamano H Kawa S Horiuchi A Unno H Furuya N Akamatsu T High serum IgG4 concentrations in patients with sclerosing pancreatitis. *N Engl J Med*. (2001) 344:732–8. 10.1056/NEJM200103083441005 11236777

[28] Stone JH Zen Y Deshpande V. IgG4-related disease. *N Engl J Med*. (2012) 366:539–51. 10.1056/NEJMra1104650 22316447

[29] Boonstra K Culver EL de Buy Wenniger LM van Heerde MJ van Erpecum KJ Poen AC Serum immunoglobulin G4 and immunoglobulin G1 for distinguishing immunoglobulin G4-associated cholangitis from primary sclerosing cholangitis. *Hepatology*. (2014) 59:1954–63. 10.1002/hep.26977 24375491 PMC4489327

[30] Nakazawa T Naitoh I Hayashi K Okumura F Miyabe K Yoshida M Diagnostic criteria for IgG4-related sclerosing cholangitis based on cholangiographic classification. *J Gastroenterol*. (2012) 47:79–87. 10.1007/s00535-011-0465-z 21947649

[31] Naitoh I Yoshida M Nakazawa T. Endoscopic diagnosis of immunoglobulin G4-related sclerosing cholangitis. *Dig Endosc*. (2025) 37:824–33. 10.1111/den.15039 40256978 PMC12333321

[32] Zhang J Chen H Ma Y Xiao Y Niu N Lin W Characterizing IgG4-related disease with ^18^F-FDG PET/CT: a prospective cohort study. *Eur J Nucl Med Mol Imaging*. (2014) 41:1624–34. 10.1007/s00259-014-2729-3 24764034 PMC4089015

[33] Nishino T Oyama H Hashimoto E Toki F Oi I Kobayashi M Clinicopathological differentiation between sclerosing cholangitis with autoimmune pancreatitis and primary sclerosing cholangitis. *J Gastroenterol*. (2007) 42:550–9. 10.1007/s00535-007-2038-8 17653651

[34] Kim MH Kwon S. Diagnostic criteria for autoimmune chronic pancreatitis. *J Gastroenterol*. (2007) 42(Suppl 18):42–9. 10.1007/s00535-007-2050-z 17520223

[35] Björnsson E Chari ST Smyrk TC Lindor K. Immunoglobulin G4 associated cholangitis: description of an emerging clinical entity based on review of the literature. *Hepatology*. (2007) 45:1547–54. 10.1002/hep.21685 17538931

[36] Oseini AM Chaiteerakij R Shire AM Ghazale A Kaiya J Moser CD Utility of serum immunoglobulin G4 in distinguishing immunoglobulin G4-associated cholangitis from cholangiocarcinoma. *Hepatology*. (2011) 54:940–8. 10.1002/hep.24487 21674559 PMC3253343

[37] Tokala A Khalili K Menezes R Hirschfield G Jhaveri KS. Comparative MRI analysis of morphologic patterns of bile duct disease in IgG4-related systemic disease versus primary sclerosing cholangitis. *AJR Am J Roentgenol*. (2014) 202:536–43. 10.2214/AJR.12.10360 24555589

[38] Radford-Smith DE Selvaraj EA Peters R Orrell M Bolon J Anthony DC A novel serum metabolomic panel distinguishes IgG4-related sclerosing cholangitis from primary sclerosing cholangitis. *Liver Int*. (2022) 42:1344–54. 10.1111/liv.15192 35129255 PMC9546203

[39] Wu Q Chang J Chen H Chen Y Yang H Fei Y Efficacy between high and medium doses of glucocorticoid therapy in remission induction of IgG4-related diseases: a preliminary randomized controlled trial. *Int J Rheum Dis*. (2017) 20:639–46. 10.1111/1756-185X.13088 28556584

[40] Kubota K Watanabe S Uchiyama T Kato S Sekino Y Suzuki K Factors predictive of relapse and spontaneous remission of autoimmune pancreatitis patients treated/not treated with corticosteroids. *J Gastroenterol*. (2011) 46:834–42. 10.1007/s00535-011-0393-y 21491208

[41] Omar D Chen Y Cong Y Dong L. Glucocorticoids and steroid sparing medications monotherapies or in combination for IgG4-RD: a systematic review and network meta-analysis. *Rheumatology*. (2020) 59:718–26. 10.1093/rheumatology/kez380 31511884

[42] Carruthers MN Topazian MD Khosroshahi A Witzig TE Wallace ZS Hart PA Rituximab for IgG4-related disease: a prospective, open-label trial. *Ann Rheum Dis*. (2015) 74:1171–7. 10.1136/annrheumdis-2014-206605 25667206

[43] Majumder S Mohapatra S Lennon RJ Piovezani Ramos G Postier N Gleeson FC Rituximab maintenance therapy reduces rate of relapse of pancreaticobiliary immunoglobulin G4-related disease. *Clin Gastroenterol Hepatol*. (2018) 16:1947–53. 10.1016/j.cgh.2018.02.049 29526692

[44] Stone JH Khosroshahi A Zhang W Della Torre E Okazaki K Tanaka Y Inebilizumab for treatment of IgG4-related disease. *N Engl J Med*. (2024) 392:1168–77. 10.1056/NEJMoa2409712 39541094

[45] Xu J Zhai J Zhao J. Pathogenic roles of follicular helper T cells in IgG4-related disease and implications for potential therapy. *Front Immunol*. (2024) 15:1413860. 10.3389/fimmu.2024.1413860 38911857 PMC11190345

